# Integrated strategy of RNA-sequencing and network pharmacology for exploring the protective mechanism of Shen-Shi-Jiang-Zhuo formula in rat with non-alcoholic fatty liver disease

**DOI:** 10.1080/13880209.2022.2106250

**Published:** 2022-09-20

**Authors:** Zheng Xu, Fan-Wei Wu, Xuan Niu, Xiao-Peng Lu, Yan-Rong Li, Shu-Ting Zhang, Jun-Zhao Ou, Xue-Mei Wang

**Affiliations:** a Liu Pai Chinese Medical Center, The Seventh Clinical Medical College of Guangzhou University of Chinese Medicine, Shenzhen, China; b School of Basic Medical Sciences, Guangzhou University of Chinese Medicine, Guangzhou, China; c Graduate School, Guangzhou University of Chinese Medicine, Guangzhou, China; d Guangdong Agriculture and Reclamation Central Hospital, Zhanjiang, Guangdong

**Keywords:** Hepatic lipid accumulation, liver function damage, hepatic fibrosis, PI3K/AKT pathway, Traditional Chinese medicine

## Abstract

**Context:**

Shen-Shi-Jiang-Zhuo formula (SSJZF) exhibits a definite curative effect in the clinical treatment of non-alcoholic fatty liver disease (NAFLD).

**Objective:**

To explore the therapeutic effect and mechanism of SSJZF on NAFLD.

**Materials and methods:**

Sprague Dawley rats were randomly divided into control, NAFLD, positive drug (12 mg/kg/day), SSJZF high-dose (200 mg/kg/day), SSJZF middle-dose (100 mg/kg/day), and SSJZF low-dose (50 mg/kg/day) groups. After daily intragastric administration of NAFLD rats for 8 weeks, lipid metabolism and hepatic fibrosis were evaluated by biochemical indices and histopathology. Then we uncovered the main active compounds and mechanism of SSJZF against NAFLD by integrating RNA-sequencing and network pharmacology, and PI3K/AKT pathway activity was verified by western blot.

**Results:**

High dose SSJZF had the best inhibitory effect on hepatic lipid accumulation and fibrosis in rats with NAFLD, which significantly down-regulated **t**otal triglycerides (58%), cholesterol (62%), aspartate aminotransferase (57%), alanine aminotransferase (41%) andγ-glutamyl transpeptidase (36%), as well as the expression of ACC (5.3-fold), FAS (12.1-fold), SREBP1C (2.3-fold), and CD36 (4.4-fold), and significantly reduced collagen deposition (67%). Then we identified 23 compounds of SSJZF that acted on 25 key therapeutic targets of NAFLD by integrating RNA-sequencing and network pharmacology. Finally, we also confirmed that high dose SSJZF increased p-PI3K/PI3K (1.6-fold) and p-AKT/AKT (1.6-fold) in NAFLD rats.

**Discussion and conclusion:**

We found for first time that SSJZF improved NAFLD in rats by activating the PI3K/Akt pathway. These findings provide scientific support for SSJZF in the clinical treatment of NAFLD and contribute to the development of new NAFLD drugs.

## Introduction

Non-alcoholic fatty liver disease (NAFLD) is the most common chronic liver disease characterised by excess lipid accumulation in liver without alcohol consumption (Brunt et al. 2015). NAFLD includes benign non-alcoholic fatty liver disease and the more severe form of non-alcoholic steatohepatitis (NASH), which can develop into cirrhosis and hepatocellular carcinoma (Cobbina and Akhlaghi 2017). NAFLD has rapidly become the most prevalent cause of liver dysfunction around the world, as lifestyles become increasingly sedentary and dietary patterns change (Zeng et al. 2020). Lifestyle modification, including weight loss and physical exercise, is still the key of NAFLD prevention and treatment (Younossi et al. 2019; Zeng et al. 2020). The current medications of NAFLD include metformin, antihyperlipidemic drugs, antioxidants, etc., which may exert serious side effects (Riley et al. 2008; Chalasani et al. 2018; Zsori et al. 2019). Thus, it is of great significance to explore more effective interventions for the treatment of NAFLD (Cobbina and Akhlaghi 2017).

Traditional Chinese medicine (TCM), as an important complementary and alternative medicine approach, has been shown efficacy and safety in treating complex diseases, including NAFLD (Qin et al. 2020; Sun et al. 2021). In TCM, the treatment of NAFLD mainly focussed on the holism of hepatoprotection, which manifests as various forms and features in mechanism, including lipid metabolism modulation and anti-fibrosis, etc. (Dai et al. 2021). As evidenced by animal and cellular studies indicated that quercetin exerts hepatoprotection against NAFLD by antihepatic steatosis or antifibrosis (Marcolin et al. 2012; Chen et al. 2021). Studies have shown that resveratrol can inhibit the accumulation of hepatic lipids and improve hepatic fibrosis for treatment of NAFLD (Izzo et al. 2021; Tejada et al. 2021). Shen-Shi-Jiang-Zhuo Formula (SSJZF) is a TCM formula consisting of *Atractylodes lancea* (Thunb.) DC. (Compositae), *Poria cocos* (Schwein) F.A. Wolf (Polyporaceae), *Magnolia officinalis* (Rehder & E. H. Wilson) N. H. Xia & C. Y. Wu (Magnoliaceae), *Rhizoma Pinelliae* (Thunb.) Breit. (Araceae), Talcum (Silicates), *Tetrapanax papyriferus* (Hook.) K. Koch (Araliaceae), *Radix Bupleuri* (Umbelliferae), *Citrus aurantium* L. (Rutaceae), *Radix Paeoniae rubra* (Ranunculaceae), *Panax notoginseng* (Burkill) F.H. Chen (Araliaceae), *Rubia cordifolia* Linn (Rubiaceae), *Fructus Broussonetiae* (Moraceae), and *Pinellia ternate* (Thunb.) Ten. ex Breitenb (Araceae). Our previous research found that SSJZF could alleviate the symptoms of NAFLD patients through improving liver function, as well as reducing the lipid levels of NAFLD patients (Lu et al. 2018). However, the underlying mechanisms and active compounds of SSJZF in the treatment of NAFLD remained unknown.

Over the past decades, with the rapid development of high-throughput analysis technologies, mRNA expression profiling has been widely used as an effective tool to explore molecular mechanisms of drug treatment of disease and uncover new therapeutic targets. For example, Cheng et al. (2019) determined that SAHA is a new treatment method for NAFLD by drug screening based on mRNA expression profile. Zhu, Li, et al. (2019) used high-throughput RNA sequencing to explore mRNA expression profiling by methionine-and-choline-deficient-induced NAFLD with or without Qinggang formula and found the protective effect of Qinggan formula on NFLDA was related to immune function. In addition, network pharmacology is a newly-developed discipline based on the large number of database resources. Network pharmacology has been widely used to explore therapeutic targets and underlying mechanisms of TCM treatment, such as discovery of TCM-derived target, prediction of pharmacological action, new multicomponent and formula discovery, etc. (Zhang et al. 2013; Jiang et al. 2015; Sun et al. 2020; Zhou et al. 2020). In brief, network pharmacology is an attractive modality to explore the association of multi-targets and multi compounds on the disease (Hopkins 2007, 2008). Network pharmacology can identify some key active ingredients and protein targets of TCM formulas involved with the disease (Chen et al. 2018; Lee et al. 2018). Furthermore, Li et al. (2021) proved that the multi-target regulation and compatibility mechanism of Gegen Qinlian Decoction on the Wnt signalling pathway by using an integrated strategy of network pharmacology and RNA-sequencing. Zhou et al. (2021) showed that base on network pharmacology combination with transcriptomics analysis to develop a novel anti-liver fibrosis new formula from Xiaoyaosan decoction, and its anti-liver fibrosis function may be related to the regulation of PI3K/AKT and JAK/STAT signalling.

In the present study, we used the rat model of high-fat-diet (HFD)-induced NAFLD to explore whether the protective effect of SSJZF on NAFLD was realised by improving hepatic lipid accumulation in clinical practice. Further, we used comprehensive analysis of mRNA-sequencing data mining, network pharmacology and bio-informatics to investigate the underlying mechanisms and effective compounds SSJZF against NAFLD, which provided theoretical basis for the application of SSJZF in clinical treatment of NAFLD.

## Methods and methods

### Drug preparation

SSJZF consists of *Atractylodes lancea* (Guangdong YuanSenTai Pharmaceutical Co., Ltd., No.190801), *Poria cocos* (Anhui XieHe ChengYao Decoction Pieces Co., Ltd., No.19111902), *Magnolia officinalis* (Traditional Chinese Medicine Decoction Pieces Factory of Guangdong Medicine Company, No.H4920711), *Rhizoma Pinelliae* (Sichuan Qianfang Traditional Chinese Medicine Co., Ltd., No.202007105), Talcum (Sichuan Qianfang Traditional Chinese Medicine Co., Ltd., No.20200701), *Tetrapanax papyriferus* (Bozhou Yonggang Decoction Pieces Factory Co., Ltd., No.200618), *Radix Bupleuri* (Traditional Chinese Medicine Decoction Pieces Factory of Guangdong Medicine Company, No.B5720214), *Citrus aurantium* (Guangdong YuanSenTai Pharmaceutical Co., Ltd., No.191001), *Radix paeoniae rubra* (Sinopharm Holdings Shenzhen Medicinal Materials Co., Ltd., No.200401), *Panax notoginseng* (Traditional Chinese Medicine Decoction Pieces Factory of Guangdong Medicine Company, No.S4420712), *Rubia cordifolia* (Inner Mongolia Yikang Pharmaceutical Co., Ltd., No19102301), and *Fructus Broussonetiae* (Anhui Boyao Qiancao Decoction Pieces Co., Ltd., No.1906054).

In accordance with the dose ratio of SSJZF (Table 1), the mixtures of crude herbs were immersed in 6 volumes of distilled water (v/w) for 60 min. After heating and refluxing for 2 h, the decoction was collected. The decoction was filtered, and the residue with 6 volumes of water refluxed again for 2 h. Finally, the water extract solution was concentrated to dryness by a rotary evaporator and stored at 4 °C for further study. The solid drugs obtained in the previous step were diluted gradient into 40, 20, and 10 mg/mL by using normal saline, and then the NAFLD rats were treated by intragastric administration according to low- (50 mg/kg/day), medium- (100 mg/kg/day), and high-dose (200 mg/kg/day). In addition, polyene phosphatidylcholine capsule group (positive drug control, PC) were diluted into 2.4 mg/mL by using normal saline, and then the NAFLD rats were treated by intragastric administration according to administration dosage (12 mg/kg/day). All drugs for intragastric administration are then diluted to the appropriate concentration when it will be used.

**Table 1.** The dosage ratio of each herb used in preparing solid SSZJF.

Drug names (Chinese name)	Manufactor	Batch number	Dose ratio of SSJZF
*Atractylodes lancea* (Cangzhu)	Guangdong YuanSenTai Pharmaceutical Co., Ltd	190801	10
*Poria cocos* (Fuling)	Anhui XieHe ChengYao Decoction Pieces Co., Ltd	19111902	15
*Magnolia officinalis* (Houpu)	Traditional Chinese Medicine Decoction Pieces Factory of Guangdong Medicine Company	H4920711	10
*Pinellia ternate* (Thunb) Breit. (Fabanxia)	Sichuan Qianfang Traditional Chinese Medicine Co., Ltd	202007105	15
Talcum (Huashi)	Guangdong Huiqun Chinese Traditional Medicine Co., Ltd	20200701	15
*Tetrapanax papyriferus* (Tongcao)	Bozhou Yonggang Decoction Pieces Factory Co., Ltd	200618	10
*Radix bupleuri* (Chaihu)	Traditional Chinese Medicine Decoction Pieces Factory of Guangdong Medicine Company	B5720214	15
*Citrus aurantium* (Zhishi)	Guangdong YuanSenTai Pharmaceutical Co., Ltd	191001	10
*Radix paeoniae rubra* (Chishao)	Sinopharm Holdings Shenzhen Medicinal Materials Co., Ltd	200401	10
*Panax notoginseng* (Sanqi)	Traditional Chinese Medicine Decoction Pieces Factory of Guangdong Medicine Company	S4420712	10
*Rubia cordifolia* (Qiancao)	Inner Mongolia Yikang Pharmaceutical Co., Ltd	19102301	10
*Fructus Broussonetiae* (Chushizi)	Anhui Boyao Qiancao Decoction Pieces Co., Ltd	1906054	30

### NAFLD rat model and drug intervention

Epidemiological and animal model studies have shown that female sex hormone oestrogen is protective against NAFLD/NASH (Hamada et al. 2000; Klair et al. 2016). To avoid the protective effects of oestrogen in female rats, male rats were chosen for the study. All the animal procedures were performed in accordance with the ‘Guide for the Care and Use of Laboratory Animals’ published by the National Institutes of Health. This study has been approved by the Commission for Bioethics and Research Ethics of Shenzhen Bao’an TCM Hospital Group (permit number: 2019013).

The NAFLD model rats were established by feeding with HFD for 4 weeks to induce NAFLD as previous described (Sheng et al. 2019). Six-week-old male SPF Sprague Dawley (SD) rats (n = 40) were purchased from Guangdong Medical Laboratory Animal Centre (Guangzhou, China). The animals were housed in cages with the temperature of 21–25 °C, humidity of 55–65%, and a 12 h dark/light cycle. All rats were allowed free access to water and food. After acclimated for 1 week, all rats were randomly divided into the following six groups (*n* = 6 per group): normal control group (NC), fed with normal diet; NAFLD group (NAFLD), fed with HFD for 16 weeks; NAFLD + polyene phosphatidylcholine capsule group (positive drug control, PC), after 8 weeks of HFD feeding, rats were orally treated with polyene phosphatidylcholine (Sigma, St. Louis, MO, USA) at a dose of 12 mg/kg/day via a lavage needle and fed on HFD feeding for 8 weeks; NAFLD + low dose SSJZF group (L-SSJZF), after 8 weeks of HFD feeding, rats were orally treated with solid SSJZF at a dose of 50 mg/kg/day via a lavage needle and fed on HFD feeding for 8 weeks; NAFLD + middle dose SSJZF group (M-SSJZF), after 8 weeks of HFD feeding, rats were orally treated with solid SSJZF at a dose of 100 mg/kg/day via a lavage needle and fed on HFD feeding for 8 weeks; NAFLD + high dose SSJZF group (H-SSJZF), after 8 weeks of HFD feeding, rats were orally treated with solid SSJZF at a dose of 200 mg/kg/day via a lavage needle and fed on HFD feeding for 8 weeks. Raw drug dose of rats was obtained according to the conversion of body surface area and man-mouse dose, and the medium dose of crude drug given to rats was the clinical dose of human. After 4 weeks intervention, rats were sacrificed to collect blood samples and live tissues samples according to previously described protocols (Feng et al. 2019) for further analysis.

### Haematoxylin and eosin (H&E) staining

For histopathological analysis, fresh liver tissues were fixed in 4% paraformaldehyde (Sigma-Aldrich, Germany) for 24 h at 4 °C, following by dehydration in alcohol gradients. And then fixed samples were processed into paraffin-embedded 4 μm sections and stained with H&E (Thermo Fisher Scientific, USA).

### Oil red O staining

For hepatic lipogenesis observation, fresh liver tissues were dehydrated with a sucrose gradient and then frozen in OCT embedding medium (Sigma-Aldrich, Germany) using liquid nitrogen. After freezing, the frozen liver tissues were sectioned at 10 μm in a freezing microtome and stained with Oil Red O (Sigma-Aldrich, Germany) in dark. Subsequently, the sections were differentiated and washed. The nucleus was stained for 3 min with haematoxylin, and sealed with glycerol gelatine for observation.

### Masson staining

The degree of hepatic fibrosis was assessed by Masson staining. Briefly, fresh liver tissue was fixed in 4% paraformaldehyde overnight, dehydrated, paraffin-embedded, and sectioned. The sections were stained using Masson kits (Service Biological Technology, Wuhan, China) according to the instructions. The collagen deposition was observed using a light microscope and analysed by the Image-Pro Plus software for relative quantification.

### Hydroxyproline (HYP) content analysis

HYP content was used to analyse by Hydroxyproline Content Assay Kit (Solarbio, Beijing, China) following the instructions. In brief, the liver samples (0.2 g) were digested with 2 mL Extract solution in 110 °C oven for 6 h. After cooling, adjust the PH to 7 with 10 mol/L NaOH constant volume to 4 mL with distilled water and then centrifuged at 16,000 rpm for 20 min. After centrifugation, the supernatant was taken and measured by visible spectrophotometry. Hydroxyproline content was corrected to liver weight and expressed as µg hydroxyproline/mg liver.

### Immunohistochemical (IHC) analysis

Immunostaining was performed to determine the subcellular localisation and expression pattern of fibrosis related protein (α-SMA, collagen I, collagen IV, or fibronectin) in HCC tissues of mice in each group. Briefly, the paraffin-embedded sections were dewaxed, rehydrated, and incubated with 3% H_2_O_2_ for blocking endogenous peroxidase. Then antigen retrieval was performed by microwave heating. After blocking non-specific antigen binding with 5% BSA at 37 °C for 1 h, the sections were incubated with a specific primary antibody against α-SMA, collagen I, collagen IV, and fibronectin (1:100 dilution, Abcam, USA) at 4 °C overnight. After incubating with the corresponding secondary antibodies at 37 °C for 1 h, the sections were stained with diaminobenzidine and counterstained with haematoxylin. Representative images were taken using an Olympus light microscope and analysed by the Image-Pro Plus software for relative quantification.

### Lipid metabolism and liver function analysis

The levels of total cholesterol (TC), triglyceride (TG), aspartate aminotransferase (AST), alanine aminotransferase (ALT), and γ-glutamyl transpeptidase (γ-GT) in serum were accessed by an automatic biochemistry analyser (Hitachi, Japan) according to the manufacturer’s instructions. In addition, the levels of TC, TG, AST, ALT, and γ-GT in liver tissues were detected using Total cholesterol assay kit (Nanjing Jiancheng Bioengineering Institute, China), Triglyceride assay kit (Nanjing Jiancheng Bioengineering Institute, China), Aspartate aminotransferase Assay Kit (Nanjing Jiancheng Bioengineering Institute, China), Alanine aminotransferase Assay Kit (Nanjing Jiancheng Bioengineering Institute, China), and γ-glutamyl transferase Assay Kit (Nanjing Jiancheng Bioengineering Institute, China), respectively.

### Real-time quantitative PCR (RT-qPCR)

Total RNA from liver tissues was extracted with Trizol (Invitrogen) according to the manufacturer’s instructions. Then cDNA synthesis was performed using PrimeScript™ RT reagent Kit with gDNA Eraser (Takara, Dalian, China). RT-qPCR was performed to determine the relative mRNA level using the TB Green^®^ Fast qPCR Mix (Takara, Dalian, China) on the ABI 7500 Fast Real-Time PCR system (Applied Biosystems). GAPDH was used as an internal control for mRNAs. The relative mRNA expression of genes was calculated through the 2^−ΔΔ^*^Ct^* method. All the primer sequences used in this study are shown in Table 2.

**Table 2.** All the primer sequences used in this study.

Genes name	Sequencing (3’–5’)	Product size (bp)
ACC	Forward: TTCCCATCCGCCTCTTCCTGAC	118
	Reverse: TGCTTGTCTCCATACGCCTGAAAC	
FAS	Forward: TGATGAAGGGCATGGTTTAGAAGTGG	111
	Reverse: TAACAGTGGTCACAGAGAGAAGCATTG	
SREBP1C	Forward: AGCAACAACAGCAGTGGCAGAG	105
	Reverse: GGTGGATGAGGGAGAGAAGGTAGAC	
CD36	Forward: CGGCGATGAGAAAGCAGAAATGTTC	91
	Reverse: TCCAACACCAAGTAAGACCATCTCAAC	
GAPDH	Forward: GTTGTCTCCTGCGACTTCA	98
	Reverse: GCCCCTCCTGTTATTATGG	

### mRNAs-sequencing and differential mRNA analysis

The next generation sequencing experiments were performed by Shanghai Biotechnology (Shanghai, China). Briefly, total RNAs from rat livers were extracted by Trizol (Thermo Scientific, USA), and the purity and integrity of RNA were detected by agarose gel electrophoresis analysis. Then, the purity of RNA (OD 260/280) was detected using Nanodrop (Thermo Scientific, USA), and the RNA concentration was accurately quantified using Qubit 2.0 (Thermo Scientific, USA). Finally, the integrity of RNA was accurately detected by Agilent 2100 (Agilent, USA). The cDNA library was constructed using NEBNext1 UltraTM RNA Library Prep Kit for Illumina (E7530, NEB, USA), and high-throughput sequencing was then performed using the HiSeqTM 2500 system (Illumina). Clean data was aligned to the rat reference genome by TopHat software and gene expression levels were assessed using fragments per kilobase of transcript per million mapped fragments (FPKM). The limma package in R was used to analyse the difference of expression matrix of sequencing data. Differentially expressed genes were identified with the threshold of *p*-value < 0.05 and |log2 FC| > 0.5. Unsupervised hierarchical clustering and the visualisation of heatmap were performed using the R platform (http://www.r-project.org/).

### Network pharmacology

#### Screening compounds and target genes of SSJZF

The Traditional Chinese Medicine Systems Pharmacology (TCMSP, https://tcmspw.com/tcmsp.php) database was used to identify the compounds in SSJZF. The screened the potential active compounds of the formula with the characteristics of oral bioavailability (OB) ≥ 30% and drug-likeness (DL) ≥ 0.18. OB indicates the drug-like nature of molecules as therapeutic agents and represents the relative amount of orally administered drug that reaches the blood circulation, shown by the convergence of the adsorption, distribution, metabolism, and excretion (ADME) process. ‘DL’ is a qualitative concept used in drug design to determine how drug-like a prospective compound is to describe and optimise pharmacokinetic and pharmaceutical properties (Zhu et al. 2019). The target genes of potential active compounds were further screened from TCMSP, and the network of potential active compounds with their target genes were constructed and visualised using Cytoscape software (version 3.7.2.).

#### Identifying the potential targets of SSJZF in NAFLD

First, the NAFLD-related target genes were gathered by the databases, including NCBI gene (https://www.ncbi.nlm.nih.gov/gene/), GeneCards (https://www.genecards.org), MalaCards (https://www.malacards.org/pages/info) (Paolacci et al. 2019), and DisGeNET (http://www.disg.enet.org) (Yu et al. 2019). Then, the overlapped genes both in NAFLD-related target genes and SSJZF target genes were displayed by Venn diagrams, which were compound-disease common targets.

#### Compound-target network construction

To study the anti-NAFLD mechanism and active compound of SSJZF, the active compounds and their potential target proteins were integrated to construct compound-target network and then were visualised using Cytoscape software (version 3.7.2.). In the network, the nodes represented the compounds and targets, and the edges represented the interactions between the targets and compounds.

### Function enrichment analysis

Based on the clusterProfiler R package (version 3.6.1; https://www.r-project.org/) installed on the Bioconductor, KEGG pathway analysis can be conducted, which can be used to explore the functional spectrum of genes or gene clusters (Chen et al. 2017). KEGG is a bioinformatics repository that contains comprehensive information related to biological pathways. The enrichment analysis results with statistical significance (*p* < 0.05) were screened out and visualised.

### Western blot analysis

Total proteins were extracted by RIPA buffer (i.e., 50 mM Tris-HCl pH 7.4, 150 mM NaCl, 2 mM EDTA, 1% NP-40, and 0.1% SDS, Thermo Fisher Scientific, USA) containing proteinase inhibitor cocktail (Roche, Germany) and phosSTOP phosphatase inhibitor cocktail (Roche, Germany) from homogenised liver tissues. Then the proteins were separated by SDS - PAGE electrophoresis, and were transferred to polyvinylidene fluoride (PVDF) membranes (Millipore, Germany). The membranes were blocked with 5% slim milk and incubated with primary antibodies including p-PI3K (ab245781, Abcam, USA; 1:1,000), PI3K (ab191606, Abcam, USA; 1:1,000), p-Akt (ab81283, Abcam, USA; 1:1,000), Akt (ab8805, Abcam, USA; 1:1,000) and GAPDH (ab8245, Abcam, USA; 1:1,000). After incubation with secondary antibodies, protein bands were visualised using an ECL detection kit (Thermo Fisher Scientific, USA). Protein expression levels were quantified by densitometry using ImageJ software (National Institutes of Health, Bethesda, MD, USA) and normalised to GAPDH (ab8245, Abcam, USA; 1:1000).

### Statistical analysis

The data were represented as the mean ± SD. All statistical analysis and plotted images were performed using GraphPad Prism 6.0 (GraphPad Software, CA). The paired or unpaired Student’s *t-*test was used for comparison between two groups and one-way ANOVA for more than two groups. The *p*-values < 0.05 were considered as statistically significant.

## Results

### SSJZF ameliorated hepatic lipid accumulation, hepatic function injury, and hepatic fibrosis in rat with NAFLD

**Figure 1.**
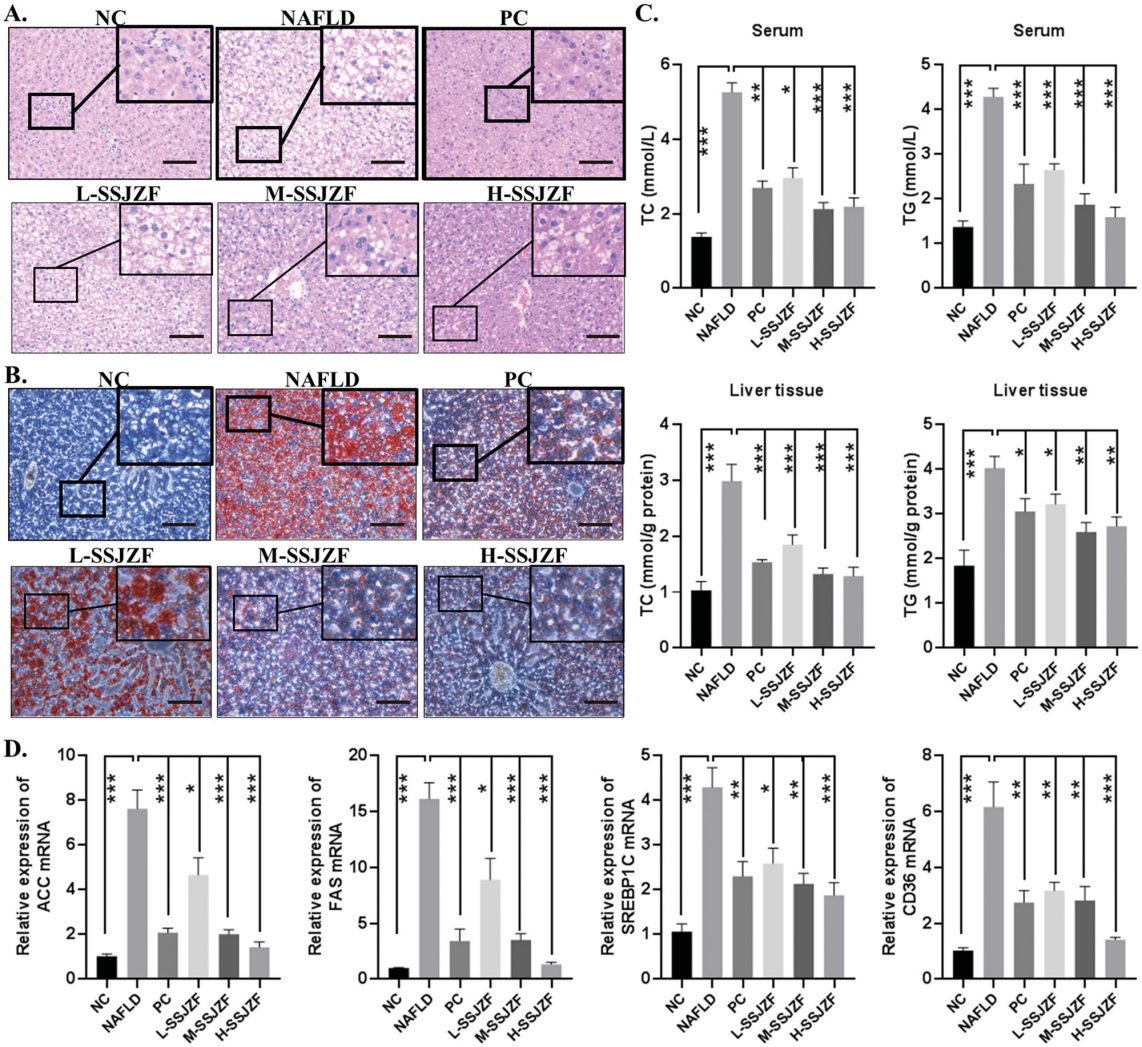
SSJZF treatment reduced hepatic lipid accumulation in rats with NAFLD (*n* = 6 per group). NAFLD: non-alcoholic fatty liver disease; SSJZF: Shen-Shi-Jiang-Zhuo formula; NC: normal control; PC: positive control; L-SSJZF: NAFLD rats treated with low dose SSJZF group; M-SSJZF: NAFLD rats treated with middle dose SSJZF group; H-SSJZF: NAFLD rats treated with high dose SSJZF group. (A) HE staining of rat liver tissues. Bar = 100 μm. (B) Oil red staining of rat liver tissues. Bar = 100 μm. (C) The content levels of TC and TG in serum and liver samples of rats were detected by an automatic biochemistry analyser. *p < 0.05, **p < 0.01, ***p < 0.001. TC, Total cholesterol; TG, Triglycerides. (D) The mRNA expression of lipid synthesis and uptake-relate genes (ACC/FAS/SREBP1C/CD36) in liver tissues were detected by RT-PCR. *p < 0.05, **p < 0.01, ***p < 0.001.

**Figure 2.**
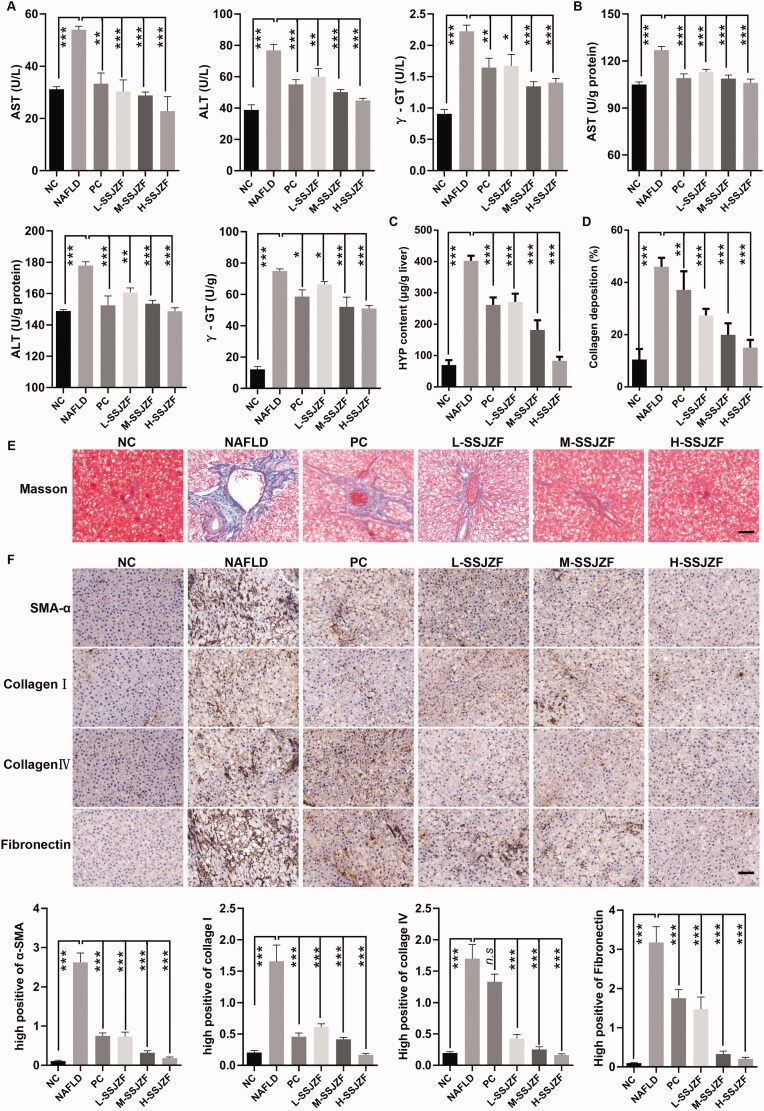
SSJZF treatment relieved hepatic function damage and fibrosis in rats with NAFLD (*n* = 6 per group). NAFLD: non-alcoholic fatty liver disease; SSJZF: Shen-Shi-Jiang-Zhuo formula; NC: normal control; PC: positive control; L-SSJZF: NAFLD rats treated with low dose SSJZF group; M-SSJZF: NAFLD rats treated with middle dose SSJZF group; H-SSJZF: NAFLD rats treated with high dose SSJZF group; AST: Aspartate aminotransferase; ALT: Alanine aminotransferase; γ-GT: γ-glutamyl transpeptidase. (A) The content levels of AST, ALT, and γ-GT in serum of rats were analysis by an automatic biochemistry analyser. *p < 0.05, **p < 0.01, ***p < 0.001. (B) The content levels of AST, ALT, and γ-GT in liver tissues of rats were analysis by an automatic biochemistry analyser. *p < 0.05, **p < 0.01, ***p < 0.001. (C) The liver fibrosis marker hydroxyproline content was measured by Hydroxyproline Content assay kit. (D) The collagen relative deposition was evaluated by Masson staining. (E) The representative Masson stain diagrams. Bar = 50 μm. (F) Immunohistochemical results showed that the reduced expressions of hepatic fibrosis by SSJZ treatment corresponded to a reduction in hepatic gene expression of fibrosis markers (α-SMA, collagen I, collagen IV, and fibronectin). Bar = 50 μm.

To confirm the efficiency of SSJZF for NAFLD treatment, rats were fed with high-fat diet (HFD) for 8 weeks to induce NAFLD and then subjected to treatment with or without SSJZF or PC by gavage for 8 weeks. The pathological changes of rat liver tissues were detected by H&E staining and Oil Red O staining (Figure 1(A,B)). Compared with NC group, cellular lipid was accumulated in the liver tissues of NAFLD rats. Low dose of SSJZF slightly reduced liver lipid deposits in the hepatocytes, while the middle and high doses of SSJZF intervention reduced lipogenesis notably in the hepatocytes. In addition, the levels of TC and TG in the serum and liver tissues of NAFLD rat models were detected by an automatic biochemistry analyser (Figure 1(C)). The results showed that the levels of TC and TG in rat with NAFLD were significantly increased compared with that of in control rats, and the effect was reduced in SSJZF-treated rats. Further, the results of RT-qPCR indicated that mRNA expression of lipid synthesis and uptake-relate genes (ACC, FAS, SREBP1C, and CD36) was significantly up-regulated in NAFLD rat model, and up-regulation of these genes were reversed by SSJZF or PC treatment (Figure 1(D)). To further investigate whether SSJZF relieved hepatic function in rats with NAFLD, we estimated the contents of AST, ALT, and γ-GT in the serum and liver tissues (Figure 2(A,B)). The results showed that the contents of AST, ALT, and γ-GT were significantly increased in NAFLD rats compared with that in control rats, and the relative indexes were reversed by middle dose or high dose of SSJZF treatment, suggesting SSJZF diminished hepatic function damage in rats with NAFLD. Finally, the degree of liver fibrosis was evaluated by Masson staining, HYP content detection kit, and immunohistochemistry. The liver fibrosis marker HYP content was significantly enhanced in liver tissues of the NAFLD rats, whereas SSJZF treatment could resist the elevated HYP content in liver tissues of the NAFLD rats (Figure 2(C)). Consistent with HYP content, Masson staining also showed that the accumulation of collagen significantly increased in liver tissues of the NAFLD rats, and the enhancement was inhibited by SSJZF treatment (Figure 2(D,E)). In addition, the results of immunohistochemistry also showed that the protein expression level of fibrosis markers (α-SMA, collagen I, collagen IV, and fibronectin) was significantly up-regulated in the NAFLD rat model, and up-regulation of these protein were reversed by SSJZF or PC treatment (Figure 2(E,F)), suggesting that SSJZF ameliorated liver fibrosis in rats with NAFLD. Altogether, our data suggested that SSJZF attenuated hepatic lipid accumulation, function damage, and fibrosis in rats with NAFLD.

### Differential mRNA expression profile of liver tissues in NAFLD rats treated with or without SSJZF

**Figure 3.**
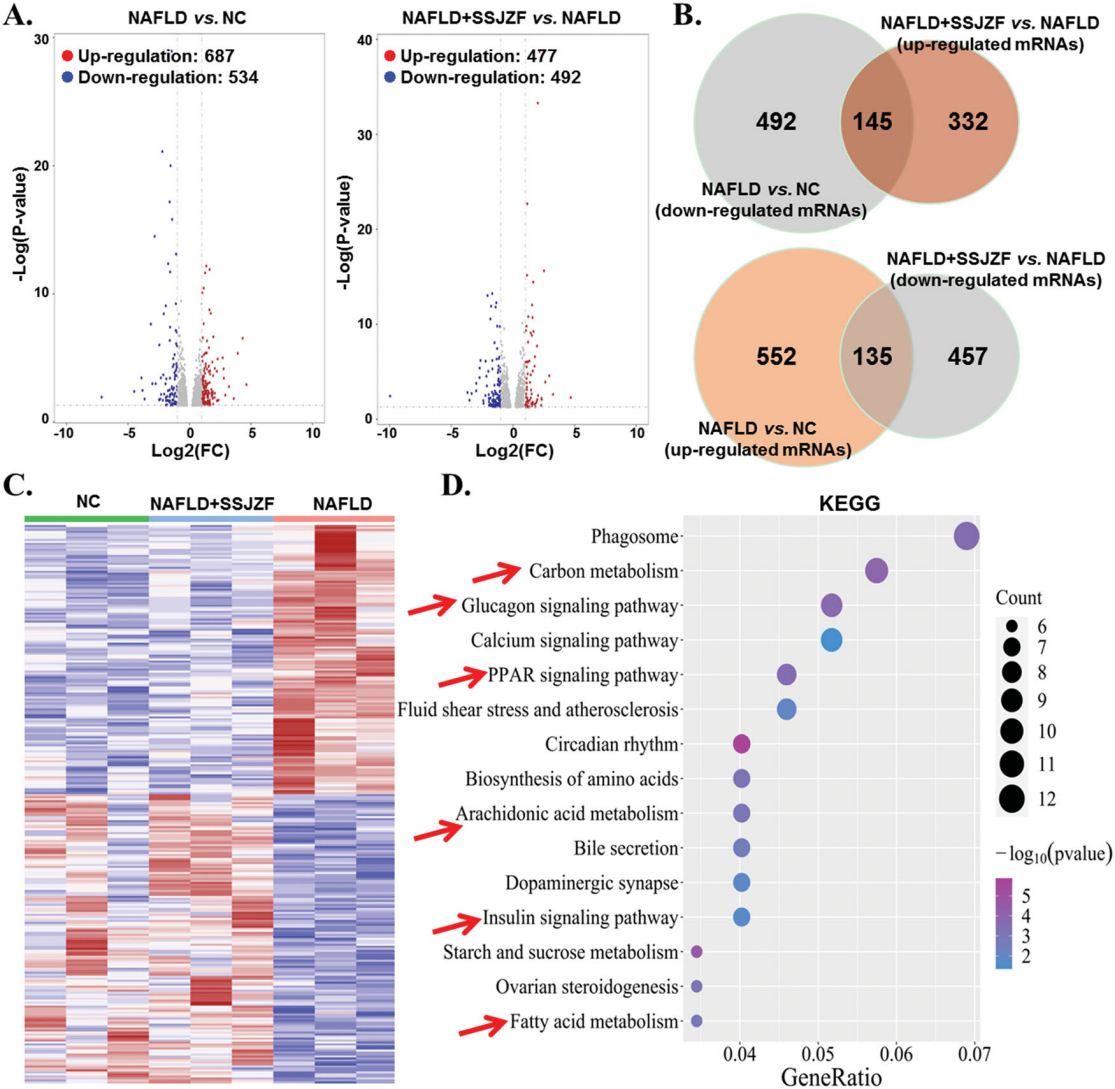
Differential mRNA expression profiles of liver tissues in NAFLD rats treated with or without SSJZF (*n* = 3 per group). NAFLD: non-alcoholic fatty liver disease; SSJZF: NAFLD rats treated with Shen-Shi-Jiang-Zhuo formula; NC: normal control. (A) Volcano plot of mRNA expression profiles between NC group and NAFLD group or NAFLD group and NAFLD + SSJZF group. (B) Venn diagram showed the intersection of differential genes between NAFLD group vs. NC group and differential genes between NAFLD + SSJZF group vs. NAFLD group. (C) Heatmap showed that 280 mRNAs of significant dysregulation in NAFLD were reversed by SSJZF treatment. (D) KEGG pathway enrichment for the 280 mRNAs of significant dysregulation.

To elucidate the therapeutic mechanism of SSJZF against NAFLD, we performed mRNAs - sequencing on livers tissues obtained from control rats, NAFLD rats, and NAFLD rats treated with SSJZF. Differentially mRNA expression was obtained with the threshold of P-value < 0.05 and |log FC| > 1. A total of 1224 differentially expressed genes were identified by comparing the NAFLD rats with the control rats, of which 687 were up-regulated and 537 were down-regulated (Figure 3(A)). A total of 969 differentially expressed genes were identified by comparing the SSJZF-treated NAFLD rats with the NAFLD rats, of which 477 were up-regulated and 492 were down-regulated (Figure 3(A)). Importantly, we found that 280 mRNAs of significantly dysregulation in NAFLD were reversed by SSJZF treatment (Figure 3(B,C)), which were enriched in 10 KEGG pathways such as fatty acid metabolism, glucagon signalling pathway, PPAR signalling pathway and arachidonic acid metabolism (Figure 3(D)). Taken together, the data suggested that a potent effect of SSJZF on a global regulation of hepatic gene expression in rats with NAFLD trending towards a normal profile, and the therapeutic effect of SSJZF on NAFLD rats might be mainly related to the regulation of lipid metabolism.

### Reconstruction and verification of SSJZF effect network based on pharmacodynamic difference gene for NAFLD

**Figure 4.**
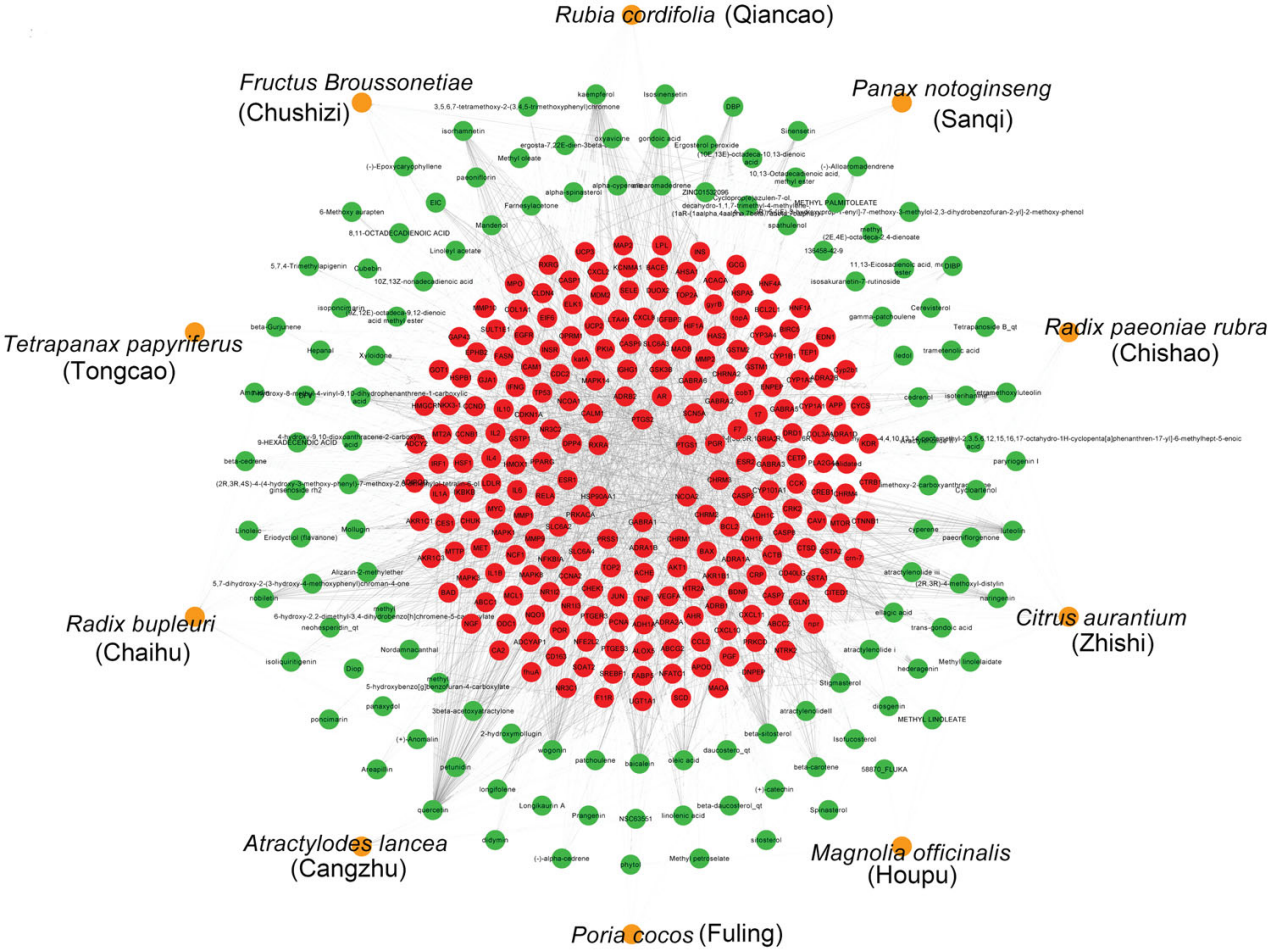
The general compounds-target network of SSJZF was constructed and visualised with Cytoscape visualisation software. The orange circles indicate the herbs of SSJZF. The green circles indicate the compounds in the herbs. The red circles indicate the potential targets of the compounds. SSJZF: Shen-Shi-Jiang-Zhuo formula.

**Figure 5.**
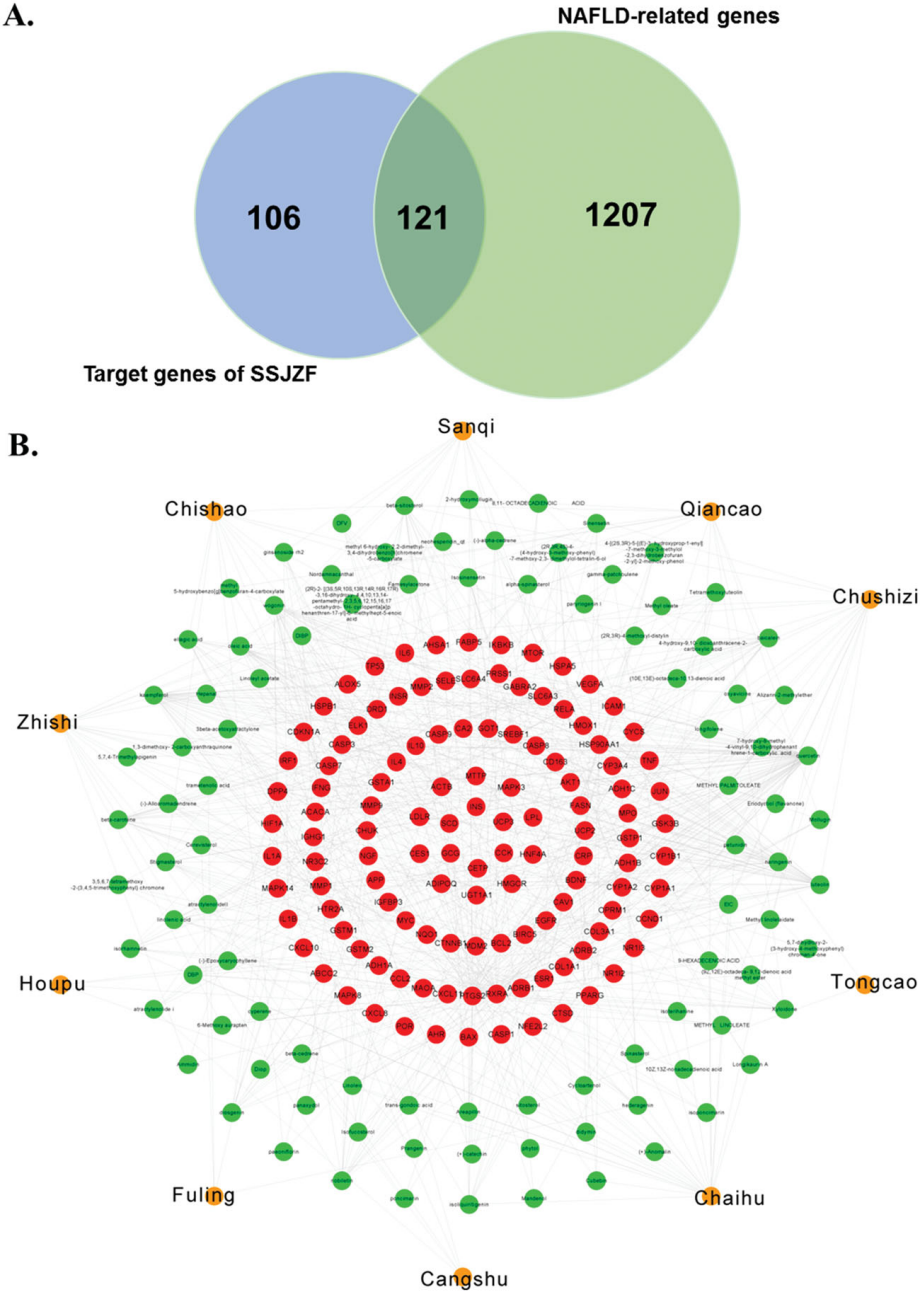
The predictive empirical compound-target network model (ECT network model) of SSJZFZ in the treatment of NAFLD. (A) 121 overlapping target genes within NAFLD-related genes and target genes of SSJZF were obtained by Venn diagram. (B) The predictive empirical compound-target network model (ECT network model) of SSJZFZ in the treatment of NAFLD was constructed by Cytoscape visualisation software. The orange circles indicate the herbs of SSJZF. The green circles indicate the compounds in the herbs. The red circles indicate the potential targets of the compounds.

**Figure 6.**
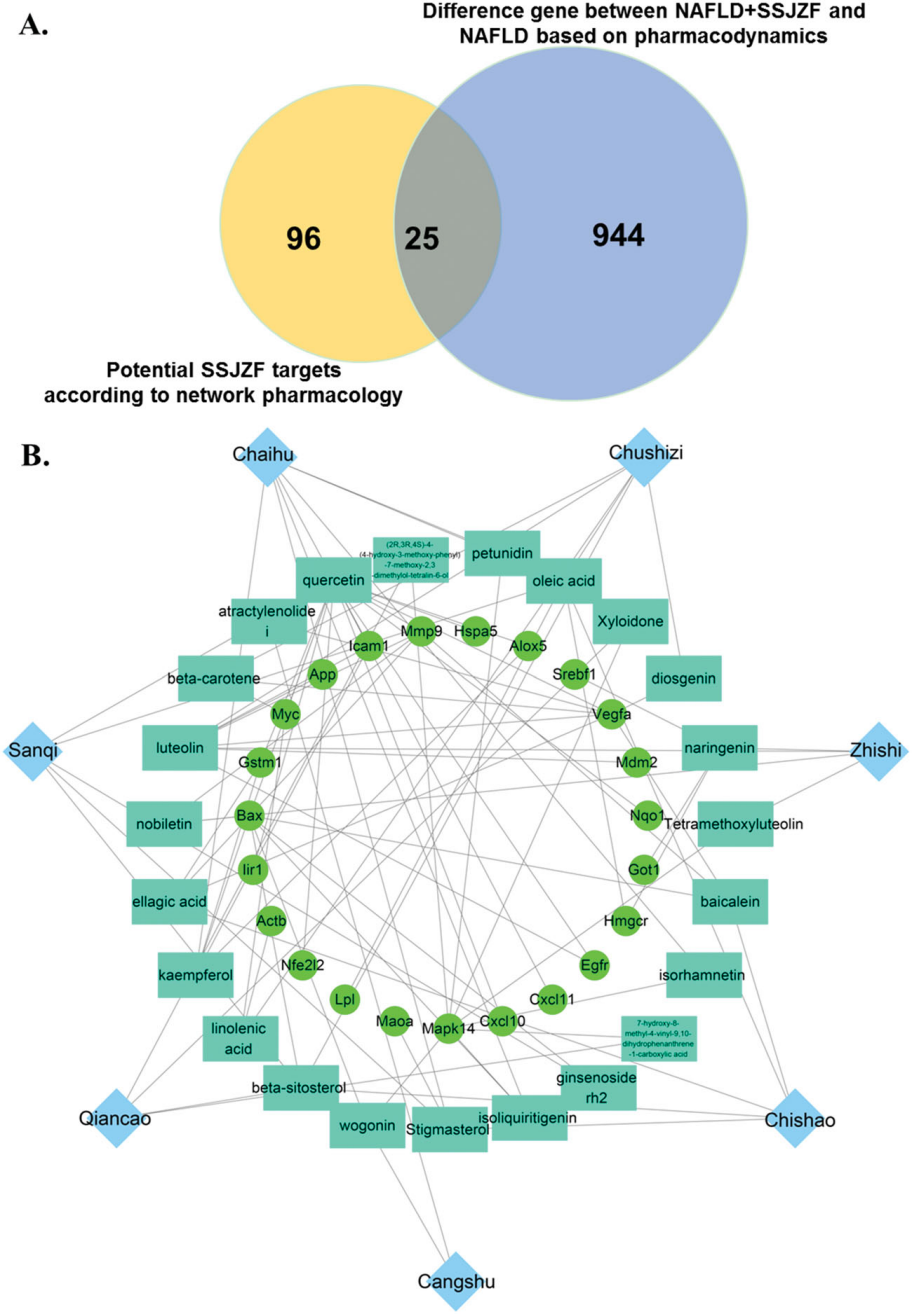
The validated compound-target network model (VCT network model) of SSJZFZ in the treatment of NAFLD. (A) Venn diagram showed 25 overlapping target genes within potential SSJZF targets according to network pharmacology and based on pharmacodynamic difference gene for NAFLD. (B) The validated compound-target network model (VCT network model) of SSJZFZ in the treatment of NAFLD was constructed by Cytoscape visualisation software. The blue rhombuses indicate the herbs of SSJZF. The cyan rectangles indicate the compounds in the herbs. The green circles indicate the potential targets of the compounds.

SSJZF consists of complexity of compounds and TCMSP was used to retrieve the potential compounds. With the limitation of OB ≥ 30% and DL ≥ 0.18, a total of 159 potential compounds of SSJZF were collected after deleting the duplicates (Table 3). The target genes of these potential compounds were further screened from TCMSP, a total of 227 target genes of these potential compounds were collected after eliminating the repetitive genes (Table 4). The SSJZF active compounds-target genes network was constructed and visualised by Cytoscape software and the compounds with the most target genes were β-sitosterol, baicalein, hederagenin, kaempferol, luteolin, oleic acid, quercetin, stigmasterol, and wogonin (Figure 4). We further screened the NAFLD-related genes using NCBI-gene, GeneCards, MalaCards and DisGeNET databases, and 1323 NAFLD-related genes were collected after eliminating the repetitive genes. Furthermore, the Venn diagram was established to clarify the relationship between SSJZF and NAFLD-related genes, and 121 overlapping target genes were obtained to be associated with NAFLD and SSJZF (Figure 5(A)). The predictive empirical compound-target network model (ECT network model) of SSJZF in the treatment of NAFLD was constructed by Cytoscape visualisation software and found that a total of 95 potential compounds in SSJZF and 121 related targets of NAFLD were collected (Figure 5(B)). On the basis this model, 969 differential genes obtained from SSJZF pharmacodynamic experiments were mapped into the ECT network model, and to reliably screen and identify 23 potential compounds of SSJZF and 25 potential target genes (Figure 6(A)). Finally, a validated compound-target network model (VCT network model) of SSJZFZ in the treatment of NAFLD was extracted and reconstructed, and the plot of the VCT network model was shown in Figure 5(B). Among the 8 compounds (quercetin, kaempferol, baicalein, ellagic acid, linolenic acid, luteolin, stigmasterol, and naringenin) were connected to more than three genes, and 7 genes (GSTEM1, MYC, ICAM, BAX, MAPK14, MMP9 and VEGFA) were regulated by more than or equal to three compounds in this network (Table 5), indicating that these compounds and genes might be key nodes in this network.

**Table 3.** Active compounds of SSJZF.

*Poria cocos*					
Herb	Mol ID	Molecule name	MW	OB (%)	DL
*Poria cocos*	MOL000291	Poricoic acid B	484.74	30.52	0.75
	MOL000290	Poricoic acid A	498.77	30.61	0.76
	MOL000273	(2*R*)-2-[(3*S*,5*R*,10*S*,13*R*,14*R*,16*R*,17*R*)-3,16-Dihydroxy-4,4,10,13,14-pentamethyl-2,3,5,6, 12,15,16,17-octahydro-1H-cyclopenta[a]phenanthren-17-yl]-6-methylhept-5-enoic acid	470.76	30.93	0.81
	MOL000280	(2*R*)-2-[(3*S*,5*R*,10*S*,13*R*,14*R*,16*R*,17*R*)-3,16-Dihydroxy-4,4,10,13,14-pentamethyl-2,3,5,6, 12,15,16,17-octahydro-1H-cyclopenta[a]phenanthren-17-yl]-5-isopropyl-hex-5-enoic acid	484.79	31.07	0.82
	MOL000289	Pachymic acid	528.85	33.63	0.81
	MOL000276	7,9(11)-Dehydropachymic acid	526.83	35.11	0.81
*Poria cocos*	MOL000296	Hederagenin	414.79	36.91	0.75
	MOL000279	Cerevisterol	430.74	37.96	0.77
	MOL000292	Poricoic acid C	482.77	38.15	0.75
	MOL000285	(2*R*)-2-[(5*R*,10*S*,13*R*,14*R*,16*R*,17*R*)-16-Hydroxy-3-keto-4,4,10,13,14-pentamethyl-1,2,5,6,1 2,15,16,17-octahydrocyclopenta[a]phenanthren-17-yl]-5-isopropyl-hex-5-enoic acid	482.77	38.26	0.82
	MOL000287	3beta-Hydroxy-24-methylene-8-lanostene-21-oic acid	470.81	38.7	0.81
	MOL000275	Trametenolic acid	456.78	38.71	0.8
	MOL000283	Ergosterol peroxide	430.74	40.36	0.81
	MOL000282	Ergosta-7,22*E*-dien-3beta-ol	398.74	43.51	0.72
	MOL000300	Dehydroeburicoic acid	453.75	44.17	0.83

*Magnolia officinalis*					
Herb	Mol ID	Molecule name	MW	OB (%)	DL
*Magnolia officinalis*	MOL000131	EIC	280.5	41.9	0.14
	MOL001442	Phytol	296.6	33.82	0.13
	MOL001641	Methyl linoleate	294.53	41.93	0.17
	MOL001889	Methyl linolelaidate	294.53	41.93	0.17
	MOL000210	Magnolol	266.36	69.19	0.15
	MOL003541	(+)-alpha-Longipinene	204.39	57.47	0.12
	MOL002003	(−)-Caryophyllene oxide	220.39	32.67	0.13
	MOL005955	Honokiol	266.36	60.67	0.15
	MOL005961	10,13-Octadecadienoic acid, methyl ester	294.53	41.93	0.17
	MOL005964	β-Panasinsene	204.39	56.28	0.12
	MOL005969	Elemol	268.49	39.27	0.11
	MOL005970	Eucalyptol	266.36	60.62	0.32
	MOL005972	Obobatol	282.36	69.45	0.18
	MOL005980	Neohesperidin	302.3	57.44	0.27
	MOL000612	(−)-alpha-Cedrene	204.39	55.56	0.1
	MOL000676	DBP	278.38	64.54	0.13
	MOL000937	58870_FLUKA	204.39	49.01	0.1

*Citrus aurantium*					
Herb	Mol ID	Molecule name	MW	OB (%)	DL
*Citrus aurantium*	MOL000131	EIC	280.5	41.9	0.14
	MOL013276	Poncirin	594.62	36.55	0.74
	MOL013277	Isosinensetin	372.4	51.15	0.44
	MOL013279	5,7,4-Trimethylapigenin	312.34	39.83	0.3
	MOL013428	Isosakuranetin-7-rutinoside	594.62	41.24	0.72
	MOL013430	Prangenin	286.3	43.6	0.29
	MOL013433	Prangenin hydrate	304.32	72.63	0.29
	MOL013435	Poncimarin	330.41	63.62	0.35
	MOL013436	Isoponcimarin	330.41	63.28	0.31
	MOL013437	6-Methoxy aurapten	328.44	31.24	0.3
	MOL013440	Citrusin B	568.63	40.8	0.71
	MOL013443	Isolimonic acid	639.02	48.86	0.18
	MOL013445	Naringenin-4-glucoside-7-rutinoside_qt	262.28	30.61	0.16
	MOL001798	Neohesperidin_qt	302.3	71.17	0.27
	MOL001803	Sinensetin	372.4	50.56	0.45
	MOL001941	Ammidin	270.3	34.55	0.22
	MOL001945	Majudin	216.2	42.21	0.13
	MOL013352	Obacunone	454.56	43.29	0.77
*Citrus aurantium*	MOL002914	Eriodyctiol (flavanone)	288.27	41.35	0.24
	MOL004328	Naringenin	272.27	59.29	0.21
	MOL005100	5,7-Dihydroxy-2-(3-hydroxy-4-methoxyphenyl)chroman-4-one	302.3	47.74	0.27
	MOL005828	Nobiletin	402.43	61.67	0.52
	MOL005849	Didymin	286.3	38.55	0.24
	MOL000006	Luteolin	286.25	36.16	0.25
	MOL007879	Tetramethoxyluteolin	342.37	43.68	0.37
	MOL009053	4-[(2*S*,3*R*)-5-[(*E*)-3-Hydroxyprop-1-enyl]-7-methoxy-3-methylol-2, 3-dihydrobenzofuran-2-yl]-2-methoxy-phenol	358.42	50.76	0.39

*Radix paeoniae rubra*					
Herb	Mol ID	Molecule name	MW	OB (%)	DL
*Radix paeoniae rubra*	MOL001002	Ellagic acid	302.2	43.06	0.43
	MOL000131	EIC	280.5	41.9	0.14
	MOL001918	Paeoniflorgenone	318.35	87.59	0.37
	MOL001921	Lactiflorin	462.49	49.12	0.8
	MOL001924	Paeoniflorin	480.51	53.87	0.79
	MOL001925	Paeoniflorin_qt	318.35	68.18	0.4
	MOL002714	Baicalein	270.25	33.52	0.21
	MOL002776	Baicalin	446.39	40.12	0.75
	MOL000358	beta-Sitosterol	414.79	36.91	0.75
	MOL000359	Sitosterol	414.79	36.91	0.75
	MOL004355	Spinasterol	412.77	42.98	0.76
	MOL000449	Stigmasterol	412.77	43.83	0.76
	MOL000492	(+)-Catechin	290.29	54.83	0.24
	MOL006990	(1*S*,2*S*,4*R*)-*trans*-2-Hydroxy-1,8-cineole-B-D-glucopyranoside	332.44	30.25	0.27
	MOL006992	(2*R*,3*R*)-4-Methoxyl-distylin	318.3	59.98	0.3

*Panax notoginseng*					
Herb	Mol ID	Molecule name	MW	OB (%)	DL
*Panax notoginseng*	MOL001494	Mandenol	308.56	42	0.19
	MOL001792	DFV	256.27	32.76	0.18
	MOL002153	1H-Cycloprop(e)azulen-7-ol, decahydro-1,1,7-trimethyl-4-methylene-, (1a*R-*(1aalpha,4aalpha,7beta,7abeta,7balpha))-	220.39	82.33	0.12
	MOL002879	Diop	390.62	43.59	0.39
	MOL000358	beta-Sitosterol	414.79	36.91	0.75
	MOL000449	Stigmasterol	412.77	43.83	0.76
	MOL005319	Ditertbutyl phthalate	278.38	43.67	0.13
	MOL005344	Ginsenoside rh2	622.98	36.32	0.56
	MOL005538	Linolenyl alcohol	264.5	42.79	0.12
	MOL000612	(−)-alpha-Cedrene	204.39	55.56	0.1
	MOL000066	Alloaromadedrene	204.39	53.46	0.1
	MOL000675	Oleic acid	282.52	33.13	0.14
	MOL007472	(9*Z*,12*E*)-Octadeca-9,12-dienoic acid methyl ester	294.53	41.93	0.17
	MOL007475	Ginsenoside f2	785.14	36.43	0.25
	MOL007486	ZINC01532096	284.15	50.74	0.15
	MOL007498	10*Z*,13*Z*-Nonadecadienoic acid	294.53	40.98	0.17
	MOL007501	Panaxydol	260.41	61.67	0.13
	MOL007503	Panaxytriol	278.43	33.76	0.13
	MOL007508	alpha-Cyperene	204.39	51.1	0.11
*Panax notoginseng*	MOL000935	Hepanal	204.39	53.83	0.1
	MOL000098	Quercetin	302.25	46.43	0.28

*Rubia cordifolia*					
Herb	Mol ID	Molecule name	MW	OB(%)	DL
*Rubia cordifolia*	MOL003283	(2*R*,3*R*,4*S*)-4-(4-Hydroxy-3-methoxy-phenyl)-7-methoxy-2,3-dimethylol-tetralin-6-ol	360.44	66.51	0.39
	MOL000358	beta-Sitosterol	414.79	36.91	0.75
	MOL000359	Sitosterol	414.79	36.91	0.75
	MOL005638	Mollugin	284.33	42.34	0.26
	MOL005869	Daucostero_qt	414.79	36.91	0.75
	MOL006139	1,3-Dimethoxy-2-carboxyanthraquinone	312.29	102.89	0.33
	MOL006141	1,3-Dihydroxy-2-hydroxymthylanthraquinone-3-O-xylosyl(1→6)-glucoside_qt	284.28	71.27	0.27
	MOL006147	Alizarin-2-methylether	254.25	32.81	0.21
	MOL006149	7-Hydroxy-8-methyl-4-vinyl-9,10-dihydrophenanthrene-1-carboxylic acid	278.32	56.99	0.27
	MOL006150	1-Acetoxy-6-hydroxy2-methylanthraquinone-3-O-α-rhamnosyl(1→4)-α-glucoside	616.67	30.74	0.64
	MOL006152	2-Carbamoyl-3-hydroxy-1,4-naphthoquinone	219.21	56.68	0.11
*Rubia cordifolia*	MOL006153	2-Hydroxymollugin	302.35	40.5	0.29
	MOL006155	4-Hydroxy-9,10-dioxoanthracene-2-carboxylic acid	266.21	45.98	0.25
	MOL006156	6-Methoxygeniposidic acid	240.28	48.32	0.11
	MOL006160	Alizarin	240.22	32.67	0.19
	MOL006162	Nordamnacanthal	268.23	53.97	0.24
	MOL006164	Pallasone	374.62	43.87	0.4
	MOL006165	Methyl 5-hydroxybenzo[*g*]benzofuran-4-carboxylate	242.24	45.7	0.17
	MOL006167	Methyl 6-hydroxy-2,2-dimethyl-3,4-dihydrobenzo[*h*]chromene-5-carboxylate	286.35	51.09	0.25
	MOL006170	Henine	270.25	77.12	0.24
	MOL006171	Rubiprasin B	498.87	35.97	0.68
	MOL006174	Xyloidone	240.27	31.61	0.18

*Fructus Broussonetiae*					
Herb	Mol ID	Molecule name	MW	OB (%)	DL
*Fructus Broussonetiae*	MOL010414	(11*E*,13*E*)-Docosa-11,13-dienoic acid	336.62	37.49	0.27
	MOL010415	11,13-Eicosadienoic acid, methyl ester	322.59	39.28	0.23
	MOL010417	8,11-Octadecadienoic acid	280.5	41.9	0.14
	MOL010421	Isoterihanine	334.37	38.05	0.77
	MOL010424	(10*E*,13*E*)-octadeca-10,13-dienoic acid	280.5	41.9	0.15
	MOL010425	Oxyavicine	347.34	53.09	0.87
	MOL010426	Methyl petroselate	296.55	31.9	0.17
	MOL001297	*trans*-Gondoic acid	310.58	30.7	0.2
	MOL000131	EIC	280.5	41.9	0.14
	MOL001494	Mandenol	308.56	42	0.19
	MOL001641	Methyl linoleate	294.53	41.93	0.17
	MOL001889	Methyl linolelaidate	294.53	41.93	0.17
	MOL002875	Methyl oleate	296.55	31.9	0.17
	MOL003578	Cycloartenol	426.8	38.69	0.78
	MOL000358	beta-Sitosterol	414.79	36.91	0.75
	MOL003717	Methyl palmitoleate	268.49	34.61	0.12
*Fructus Broussonetiae*	MOL000432	Linolenic acid	278.48	45.01	0.15
	MOL004683	Methyl (2*E*,4*E*)-octadeca-2,4-dienoate	294.53	38.77	0.17
	MOL005030	Gondoic acid	310.58	30.7	0.2
	MOL005440	Isofucosterol	412.77	43.78	0.76
	MOL000546	Diosgenin	414.69	80.88	0.81
	MOL005736	Cyperene	204.39	51.87	0.11
	MOL005961	10,13-Octadecadienoic acid, methyl ester	294.53	41.93	0.17
	MOL000006	Luteolin	286.25	36.16	0.25
	MOL000675	Oleic acid	282.52	33.13	0.14
	MOL008162	Cyclosativene	204.39	38.18	0.15
	MOL002773	beta-Carotene	536.96	37.18	0.58

*Tetrapanax papyriferus*					
Herb	Mol ID	Molecule name	MW	OB (%)	DL
*Tetrapanax papyriferus*	MOL008025	Tetrapanoside B_qt	428.77	40.93	0.79
	MOL000359	Sitosterol	414.79	36.91	0.75
	MOL008020	Paryriogenin I	422.71	45.26	0.79
	MOL008006	Paryriogenin A	466.72	41.41	0.76

*Radix Bupleuri*					
Herb	Mol ID	Molecule name	MW	OB (%)	DL
*Radix Bupleuri*	MOL001179	(−)-Alloaromadendrene	204.39	54.04	0.1
	MOL000131	EIC	280.5	41.9	0.14
	MOL001645	Linoleyl acetate	308.56	42.1	0.2
	MOL001789	Isoliquiritigenin	256.27	85.32	0.15
	MOL002335	beta-Gurjunene	204.39	51.36	0.1
	MOL002776	Baicalin	446.39	40.12	0.75
	MOL000449	Stigmasterol	412.77	43.83	0.76
	MOL000354	Isorhamnetin	316.28	49.6	0.31
	MOL000422	Kaempferol	286.25	41.88	0.24
	MOL004598	3,5,6,7-Tetramethoxy-2-(3,4,5-trimethoxyphenyl)chromone	432.46	31.97	0.59
	MOL004609	Areapillin	360.34	48.96	0.41
	MOL013187	Cubebin	356.4	57.13	0.64
	MOL004624	Longikaurin A	348.48	47.72	0.53
	MOL004628	Octalupine	264.41	47.82	0.28
	MOL004642	Saikosaponin t	813.15	34.46	0.13
	MOL004644	Sainfuran	286.3	79.91	0.23
	MOL004648	Troxerutin	346.56	31.6	0.28
	MOL004653	(+)-Anomalin	426.5	46.06	0.66
*Radix Bupleuri*	MOL004658	Cedrenol	220.39	108.56	0.12
	MOL004677	Ledol	222.41	82.78	0.12
	MOL004679	Longifolene	204.39	39.49	0.11
	MOL004683	Methyl (2*E*,4*E*)-octadeca-2,4-dienoate	294.53	38.77	0.17
	MOL004702	Saikosaponin c_qt	472.78	30.5	0.63
	MOL004706	Spathulenol	220.39	80.01	0.12
	MOL004718	alpha-Spinasterol	412.77	42.98	0.76
	MOL004720	beta-Cedrene	204.39	54.35	0.11
	MOL004728	gamma-Patchoulene	204.39	54.63	0.11
	MOL000490	Petunidin	317.29	30.05	0.31
	MOL000057	DIBP	278.38	49.63	0.13
	MOL000675	Oleic acid	282.52	33.13	0.14
	MOL000676	DBP	278.38	64.54	0.13
	MOL000878	Farnesylacetone	262.48	37.84	0.1
	MOL000098	Quercetin	302.25	46.43	0.28
	MOL000474	(−)-Epoxycaryophyllene	220.39	35.94	0.13
	MOL000749	Linoleic	280.5	41.9	0.14
	MOL000937	58870_FLUKA	204.39	49.01	0.1
*Radix Bupleuri*	MOL001312	9-Hexadeceoic acid	254.46	35.78	0.1
	MOL002338	136458-42-9	220.39	80.86	0.12

*Atractylodes lancea*					
Herb	Mol ID	Molecule name	MW	OB (%)	DL
*Atractylodes lancea*	MOL000164	Atractylone	216.35	33.91	0.13
	MOL000173	Wogonin	284.28	30.68	0.23
	MOL000175	Cyperene	204.39	51.1	0.11
	MOL000043	Atractylenolide i	230.33	37.37	0.15
	MOL000178	Atractylenolide iii	248.35	31.66	0.17
	MOL000179	2-Hydroxyisoxypropyl-3-hydroxy-7-isopentene-2,3-dihydrobenzofuran-5-carboxylic	306.39	45.2	0.2
	MOL000180	Aractylenolide II	232.35	46.2	0.15
	MOL000181	Atractylenolide III	248.35	31.15	0.17
	MOL000184	NSC63551	412.77	39.25	0.76
	MOL000186	Stigmasterol 3-*O*-beta-D-glucopyranoside_qt	412.77	43.83	0.76
	MOL000187	Butenolide B	234.32	61	0.15
	MOL000188	3β-Acetoxyatractylone	274.39	40.57	0.22
	MOL000194	Patchoulene	204.39	51.71	0.11
	MOL000044	AtractylenolideII	232.35	47.5	0.15
	MOL000085	beta-Daucosterol_qt	414.79	36.91	0.75
	MOL000088	beta-Sitosterol 3-*O*-glucoside_qt	414.79	36.91	0.75
*Atractylodes lancea*	MOL000092	Daucosterin_qt	414.79	36.91	0.76
	MOL000094	Daucosterol_qt	414.79	36.91	0.76

**Table 4.** Target genes of SSJZF active compounds.

Gene name	Protein name
HTR2A	5-Hydroxytryptamine 2A receptor
ADRA1A	alpha-1A Adrenergic receptor
ADRA1B	alpha-1B Adrenergic receptor
ADRB1	beta-1 Adrenergic receptor
ADRB2	beta-2 Adrenergic receptor
DRD1	Dopamine D1 receptor
GABRA3	gamma-Aminobutyric-acid receptor alpha-3 subunit
GABRA1	Gamma-Aminobutyric acid receptor subunit alpha-1
CHRM1	Muscarinic acetylcholine receptor M1
CHRM2	Muscarinic acetylcholine receptor M2
CHRM3	Muscarinic acetylcholine receptor M3
CHRNA2	Neuronal acetylcholine receptor subunit alpha-2
NCOA2	Nuclear receptor coactivator 2
RXRA	Retinoic acid receptor RXR-alpha
SLC6A3	Sodium-dependent dopamine transporter
SLC6A2	Sodium-dependent noradrenaline transporter
ADH1B	Alcohol dehydrogenase 1B
ADH1C	Alcohol dehydrogenase 1C
Gene name	Protein name
GABRA2	Gamma-aminobutyric-acid receptor alpha-2 subunit
PTGS2	Prostaglandin G/H synthase 2
ACHE	Acetylcholinesterase
DPP4	Dipeptidyl peptidase IV
GABRA6	Gamma-aminobutyric-acid receptor subunit alpha-6
TOP2	DNA topoisomerase II
CALM1	Calmodulin
katA	Catalase
ESR1	Oestrogen receptor
HSP90AA1	Heat shock protein HSP 90
HAS2	Hyaluronan synthase 2
PRKACA	mRNA of PKA Catalytic Subunit C-alpha
PTGS1	Prostaglandin G/H synthase 1
NR3C2	Mineralocorticoid receptor
AR	Androgen receptor
CA2	Carbonic anhydrase II
CA2	Carbonic anhydrase II
F7	Coagulation factor VII
CCNA2	Cyclin-A2
ESR2	Oestrogen receptor beta
GSK3B	Glycogen synthase kinase-3 beta
IGHG1	Ig gamma-1 chain C region
MAPK14	Mitogen-activated protein kinase 14
PPARG	Peroxisome proliferator activated receptor gamma
Gene name	Protein name
CHEK1	Serine/threonine-protein kinase Chk1
SCN5A	Sodium channel protein type 5 subunit alpha
PRSS1	Trypsin-1
OPRM1	Mu-type opioid receptor
SLC6A4	Sodium-dependent serotonin transporter
AKR1B1	Aldose reductase
PKIA	cAMP-dependent protein kinase inhibitor alpha
ADRA1D	Alpha-2C adrenergic receptor
NCOA1	Nuclear receptor coactivator 1
CYP101A1	Cytochrome P450-cam
fhuA	Ferrichrome-iron receptor
MAOB	Amine oxidase [flavin-containing] B
IL1B	Interleukin-1 beta
IL6	Interleukin-6
PGF	Placenta growth factor
TNF	Tumour necrosis factor
GRIA2	Glutamate receptor 2
FABP5	Fatty acid-binding protein, epidermal
NFATC1	Nuclear factor of activated T-cells, cytoplasmic 1
APOD	Apolipoprotein D
BAX	Apoptosis regulator BAX
Gene name	Protein name
BCL2	Apoptosis regulator Bcl-2
AHR	Aryl hydrocarbon receptor
CASP3	Caspase-3
CRK2	Cell division control protein 2 homolog
TP53	Cellular tumour antigen p53
CYCS	Cytochrome c
EGLN1	Egl nine homolog 1
CCNB1	G2/mitotic-specific cyclin-B1
HIF1A	Hypoxia-inducible factor 1-alpha
IGFBP3	Insulin-like growth factor II
MMP9	Matrix metalloproteinase-9
MPO	Myeloperoxidase
AKT1	RAC-alpha serine/threonine-protein kinase
RELA	Transcription factor p65
MMP2	72 kDa type IV collagenase
CASP7	Caspase-7
CASP8	Caspase-8
CASP9	Caspase-9
CTNNB1	Catenin beta-1
CAV1	Caveolin-1
CYP1A2	Cytochrome P450 1A2
Cyp2b1	Cytochrome P450 2B1
CYP3A4	Cytochrome P450 3A4
GJA1	Gap junction alpha-1 protein
HMOX1	Heme oxygenase 1
MMP1	Interstitial collagenase
MYC	Myc proto-oncogene protein
validated	Serum albumin
Gene name	Protein name
MMP10	Stromelysin-2
JUN	Transcription factor AP-1
PGR	Progesterone receptor
GABRA5	Gamma-aminobutyric-acid receptor alpha-5 subunit
MAP2	Microtubule-associated protein 2
CHRM4	Muscarinic acetylcholine receptor M4
NR3C1	Glucocorticoid receptor
ABCC2	Canalicular multispecific organic anion transporter 1
CDKN1A	Cyclin-dependent kinase inhibitor 1
PLA2G4A	Cytosolic phospholipase A2
FASN	Fatty acid synthase
NR1I2	Nuclear receptor subfamily 1 group I member 2
MTOR	Serine/threonine-protein kinase mTOR
cobT	Nicotinate-nucleotide--dimethylbenzimidazole phosphoribosyltransferase
CDC2	Cell division protein kinase 2
GSTA1	Glutathione *S*-transferase A1
GSTA2	Glutathione *S*-transferase A2
GSTM1	Glutathione *S*-transferase Mu 1
GSTM2	Glutathione *S*-transferase Mu 2
GSTP1	Glutathione *S*-transferase P
CXCL8	Interleukin-8
NFKBIA	NF-kappa-B inhibitor alpha
CASP1	Caspase-1
IFNG	Interferon gamma
Gene name	Protein name
ADCYAP1	Pituitary adenylate cyclase-activating polypeptide
ADH1A	Alcohol dehydrogenase 1A
npr	Bacillolysin
SELE	E-selectin
F11R	Junctional adhesion molecule A
MT2A	Metallothionein-2
NCF1	Neutrophil cytosol factor 1
KCNMA1	Calcium-activated potassium channel subunit alpha 1
AHSA1	Activator of 90 kDa heat shock protein ATPase homolog 1
AKR1C3	Aldo-keto reductase family 1 member C3
ALOX5	Arachidonate 5-lipoxygenase
CYP1A1	Cytochrome P450 1A1
CYP1B1	Cytochrome P450 1B1
IKBKB	Inhibitor of nuclear factor kappa-B kinase subunit beta
ICAM1	Intercellular adhesion molecule 1
MAPK8	Mitogen-activated protein kinase 8
NR1I3	Nuclear receptor subfamily 1 group I member 3
ADRA2A	Alpha-2A adrenergic receptor
ADRA2B	Alpha-2B adrenergic receptor
ACTB	Actin, cytoplasmic 1
HNF1A	Hepatocyte nuclear factor 1-alpha
HNF4A	Hepatocyte nuclear factor 4-alpha
UCP2	Mitochondrial uncoupling protein 2
Gene name	Protein name
PCNA	Proliferating cell nuclear antigen
INSR	Insulin receptor
RXRG	Retinoic acid receptor RXR-gamma
ADCY2	Adenylate cyclase type 2
APP	Amyloid beta A4 protein
BIRC5	Baculoviral IAP repeat-containing protein 5
BCL2L1	Bcl-2-like protein 1
CD40LG	CD40 ligand
topA	DNA topoisomerase 1
TOP2A	DNA topoisomerase 2-alpha
MDM2	E3 ubiquitin-protein ligase Mdm2
EGFR	Epidermal growth factor receptor
CCND1	G1/S-specific cyclin-D1
MET	Hepatocyte growth factor receptor
MCL1	Induced myeloid leukaemia cell differentiation protein Mcl-1
IL10	Interleukin-10
IL2	Interleukin-2
IL4	Interleukin-4
MAPK1	Mitogen-activated protein kinase 1
PTGES3	Prostaglandin E synthase
GOT1	Aspartate aminotransferase, cytoplasmic
HMGCR	3-Hydroxy-3-methylglutaryl-coenzyme A reductase
ADIPOQ	Adiponectin
AKR1C1	Aldo-keto reductase family 1 member C1
BAD	Bcl2 antagonist of cell death
Gene name	Protein name
CES1	Liver carboxylesterase 1
LDLR	Low-density lipoprotein receptor
MTTP	Microsomal triglyceride transfer protein large subunit
MAPK3	Mitogen-activated protein kinase 3
ABCC1	Multidrug resistance-associated protein 1
SOAT2	Sterol *O*-acyltransferase 2
SREBF1	Sterol regulatory element-binding protein 1
UGT1A1	UDP-glucuronosyltransferase 1-1
CREB1	Cyclic AMP-responsive element-binding protein 1
EPHB2	Ephrin type-B receptor 2
CD163	Scavenger receptor cysteine-rich type 1 protein M130
SCD	Acyl-CoA desaturase
NTRK2	BDNF/NT-3 growth factors receptor
DNPEP	Aspartyl aminopeptidase
BDNF	Brain-derived neurotrophic factor
CRP	C-reactive protein
CITED1	Cbp/p300-interacting transactivator 1
crn-7	Cell-death-related nuclease 7
CCK	Cholecystokinin
CETP	Cholesteryl ester transfer protein
EDN1	Endothelin-1
GCG	Glucagon
ENPEP	Glutamyl aminopeptidase
INS	Insulin
LPL	Lipoprotein lipase
Gene name	Protein name
UCP3	Mitochondrial uncoupling protein 3
GAP43	Neuromodulin
TEP1	Telomerase protein component 1
NGF	Beta-nerve growth factor
EIF6	Eukaryotic translation initiation factor 6
HSPA5	78 kDa glucose-regulated protein
ACACA	Acetyl-CoA carboxylase 1
ABCG2	ATP-binding cassette sub-family G member 2
CCL2	C-C motif chemokine 2
CXCL10	C-X-C motif chemokine 10
CXCL11	C-X-C motif chemokine 11
CXCL2	C-X-C motif chemokine 2
CTSD	Cathepsin D
COL1A1	Collagen alpha-1(I) chain
COL3A1	Collagen alpha-1(III) chain
gyrB	DNA gyrase subunit B
DUOX2	Dual oxidase 2
SULT1E1	Oestrogen sulfotransferase
ELK1	ETS domain-containing protein Elk-1
HSF1	Heat shock factor protein 1
HSPB1	Heat shock protein beta-1
NKX3-1	Homeobox protein Nkx-3.1
CHUK	Inhibitor of nuclear factor kappa-B kinase subunit alpha
IRF1	Interferon regulatory factor 1
IL1A	Interleukin-1 alpha
NQO1	NAD(P)H dehydrogenase [quinone] 1
Gene name	Protein name
POR	NADPH--cytochrome P450 reductase
NFE2L2	Nuclear factor erythroid 2-related factor 2
ODC1	Ornithine decarboxylase
PTGER3	Prostaglandin E2 receptor EP3 subtype
CLDN4	Claudin-4
MAOA	Amine oxidase [flavin-containing] A
CTRB1	Chymotrypsinogen B
LTA4H	Leukotriene A-4 hydrolase
BACE1	beta-Secretase
PRKCD	Protein kinase C delta type
KDR	Vascular endothelial growth factor receptor 2

**Table 5.** The validated compound-target network model (VCT network model) of SSJZFZ in the treatment of NAFLD was identified 23 compounds of SSJZF and 25 potential target genes.

Herbs	Compounds	Targets
Cangshu	Wogonin	Bax, Mapk14
	Atractylenolide i	Vegfa
Chaihu	Quercetin	Alox5, Bax, Cxcl10, Cxcl11, Egfr, Gstm1, Hspa5, Icam1, Irf1, Mmp9, Myc, Nfe2l2, Nqo1
	Kaempferol	Alox5, Bax, Gstm1, Icam1
	Isoliquiritigenin	Bax, Mapk14
	Oleic acid	Hmgcr, Lpl
	Stigmasterol	Maoa
	isorhamnetin	Mapk14
	Petunidin	Mapk14
Chishao	Baicalein	Bax, Mmp9, Vegfa
	beta-Sitosterol	Bax
	Ellagic acid	Gstm1, Mmp9, Vegfa
	Oleic acid	Hmgcr, Lpl
	Stigmasterol	Maoa
Chushizi	Linolenic acid	Actb, Myc, Vegfa
	Luteolin	App, Egfr, Icam1, Mdm2, Mmp9, Vegfa
	beta-Sitosterol	Bax
	Oleic acid	Hmgcr, Lpl
	beta-Carotene	Myc
	Diosgenin	Vegfa
Qiancao	beta-Sitosterol	Bax
	7-Hydroxy-8-methyl-4-vinyl-9,10-dihydrophenanthrene-1-carboxylic acid	Mapk14
	Xyloidone	Mapk14
	(2*R*,3*R*,4*S*)-4-(4-Hydroxy-3-methoxy-phenyl)-7-methoxy-2,3-dimethylol-tetralin-6-ol	Mapk14
Sanqi	Quercetin	Alox5, Bax, Cxcl10, Cxcl11, Egfr, Gstm1, Hspa5, Icam1, Irf1, Mmp9, Myc, Nfe2l2, Nqo1
	beta-Sitosterol	Bax
	Ginsenosiderh2	Bax
	Oleic acid	Hmgcr, Lpl
	Stigmasterol	Maoa, Egfr, Icam1, Mdm2, Mmp9, Vegfa
Zhishi	Luteolin	App, Mmp9
	Nobiletin	Bax
	Naringenin	Got1, Hmgcr, Srebf1
	Tetramethoxyluteolin	Mapk14

**Figure 7.**
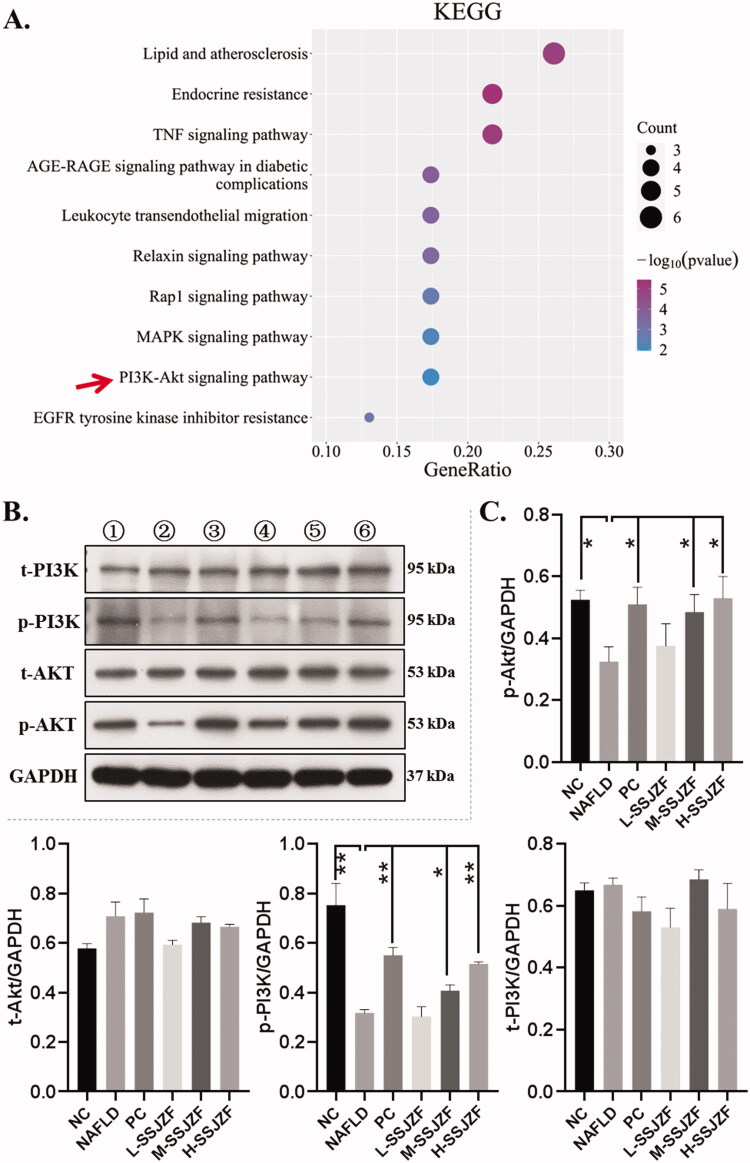
SSJZF enhanced PI3K/Akt signalling pathway activation in rat with NAFLD. NAFLD: non-alcoholic fatty liver disease; SSJZF: Shen-Shi-Jiang-Zhuo formula; NC: normal control; PC: positive control; L-SSJZF: NAFLD rats treated with low dose SSJZF group; M-SSJZF: NAFLD rats treated with middle dose SSJZF group; H-SSJZF: NAFLD rats treated with high dose SSJZF group. A. KEGG pathway enrichment of the intersection 25 different genes. B/C. The expression of total PI3K (t-PI3K), phosphorylated PI3K (p-PI3K), total Akt (t-Akt) and phosphorylated Akt (p-Akt) in liver tissues was detected by Western blots. *p < 0.05, **p < 0.01.

To further examine the signalling pathways and functions of these target genes, we performed functional enrichment analysis using KEGG pathways. The result indicated that 25 genes were mainly enriched in PI3K/Akt signalling pathway, endocrine resistance, TNF signalling pathway, lipid and atherosclerosis, Rap1 signalling pathway, MAPK signalling pathway, etc. (Figure 7(A)). Taken together, our data suggested that the treatment of SSJZF on NAFLD might be achieved through multi-compounds, multi-targets, and multi-pathways.

### SSJZF enhanced PI3K/Akt signalling pathway activity in liver tissues of NAFLD rats

PI3K/Akt signalling pathway has also been demonstrated to play a critical role in the regulation of liver lipid metabolism in NAFLD (Wang et al. 2014; Zeng et al. 2014; Wang et al. 2019). The comprehensive analysis of network pharmacology and sequencing in the early stage found that PI3K signalling pathway was involved in the therapeutic effect of SSJZF on NAFLD (Figure 7(A)). Here, in order to confirm whether SSJZF regulates PI3K/Akt signalling pathway in liver tissue of NAFLD rats, western blot analyses were performed to verify the PI3K/Akt signalling pathway activation in NAFLD rats with or without SSJZF treatment (Figure 7(B,C)). Compared with the control group, the phosphorylation levels of PI3K and AKT was significantly decreased in liver tissues of NAFLD rats, but the total protein of PI3K and AKT was not remarkably altered, indicating PI3K/Akt signalling pathway was restrained in liver tissues of NAFLD rats. While the restraint of PI3K/Akt signalling pathway in liver tissues of NAFLD rats was reversed by treatment of PC or SSJZF, indicating SSJZF enhanced PI3K/Akt signalling pathway activity in liver tissues of NAFLD rats. And the efficiency of high dose of SSJZF is more significant than PC in PI3K/Akt signalling pathway activation. Taken together, our data indicated that SSJZF might alleviate hepatic lipid accumulation in NAFLD rat model via activating the PI3K/Akt signalling pathway.

## Discussion

NAFLD, currently most common form of chronic liver disease in both adults and children worldwide, is associated with hepatic lipids metabolism (Ipsen et al. 2018; Gjorgjieva et al. 2019; Reimer et al. 2020). Over the last decades, great studies have been dedicated to the understanding of the pathogenesis of NAFLD, but there are currently no effective drugs to treat NAFLD (Gao et al. 2020). In this study, our results revealed that SSJZF ameliorated high-fat diet-induced NAFLD in rats. Further, the comprehensive analysis of mRNA-sequencing and network pharmacology was used to uncover the main active ingredients and protective mechanisms of SSJZF in NAFLD.

The traditional Chinese medicine SSJZF has been clinically used to treat NAFLD for a long time in China. In our previous study, we indicated that SSJZF had higher clinical therapeutic efficacy on NAFLD (Lu et al. 2018). However, preclinical experimental evidence supporting its efficacy is lacking. Our results revealed for first time that SSJZF improve abnormal lipid metabolism, liver function injury, and hepatic fibrosis in rats with NAFLD, suggesting that SSJZF ameliorated HFD-induced NAFLD in rats. The results of this study are consistent with our preliminary clinical study. Liver fibrosis, also one of the important characteristics of NAFLD, is a necessary progression for NAFLD to develop into non-alcoholic steatohepatitis, cirrhosis or hepatocellular carcinoma (HCC), and is also a reversible pathological stage that can effectively intervene and prevent NAFLD (Campos-Murguía et al. 2020). Animal experiment showed that apoptosis signal-regulating kinase 1 (ASK1) depletion increased hepatic steatosis and liver fibrosis, and ASK1 may be a novel therapeutic target to the hepatic fibrosis of NAFLD (Challa et al. 2019). In addition, studies have been carried out on the therapeutic effect of Traditional Chinese medicine in NAFLD fibrosis. For example, polydatin alleviate liver fibrosis and relieves hepatic fat accumulation in rats with NAFLD (Li et al. 2018). Silybin prevented hepatic steatosis and fibrosis in NASH mice by inhibiting Nf-κB Pathway (Takatani et al. 2020). Our results indicated that SSJZF reduced the hepatic fibrosis in rats with NAFLD. Studies have shown that quercetin can ameliorate tissue fibrosis in a variety of diseases, including hepatic fibrosis (Wu et al. 2017), and this study indirectly supports our findings.

To further explore the protective mechanism of SSJZF in the treatment of HFD-induced NAFLD in rats, we performed mRNA-sequencing analysis. Our data indicated that 280 mRNAs of significantly dysregulation in NAFLD were reversed by SSJZF treatment, which were enriched 10 KEGG, such as arachidonic acid metabolism and fatty acid metabolism. Recent studies have shown that the abnormality of hepatic lipid metabolism (such as fatty acid metabolism and arachidonic acid metabolism) is a key factor leading to the occurrence and development of NAFLD (Pei et al. 2020). Sztolsztener et al. (2020) suggested that arachidonic acid is an early indicator of inflammation in the development of NAFLD. Furthermore, we identified 23 active compounds of SSJZF in NAFLD base on mRNAs-sequencing combined with network pharmacology. Among the active compounds of SSJZF, quercetin, β-carotene, luteolin, stigmasterol, and diosgenin have been proved to alleviate NAFLD. For example, quercetin is a flavonoid which takes part in various processes, such as anti-inflammatory and antioxidative (Wang et al. 2020). It is proved that the combination of quercetin and DHA-rich fish oil may partly alleviate NAFLD by reducing oxidative stress, suppressing fatty acid synthesis, ameliorating inflammation (Xu et al. 2019; Yang et al. 2019). A cross-sectional study showed that β-carotene to retinol ratio is associated with the severity of NAFLD (Kimura et al. 2020). It is reported that diosgenin ameliorated free fatty acid-induced NAFLD via AMPK/ACC pathway (Fang et al. 2019). Many studies have shown that luteolin alone and combination therapy may be the treatment of NAFLD development (Sagawa et al. 2015; Zhu et al. 2020; Sun et al. 2021). Moreover, Studies have shown that stigmasterol alters lipid metabolism and alleviates NAFLD in mice fed a high-fat western-style diet (Feng, Dai, et al. 2018; Feng, Gan, et al. 2018). Furthermore, our previous research has proved that SSJZF significantly reduces the process of NAFLD clinically (Lu et al. 2018). Taking the results into consideration, we suggested that SSJZF may reduce NAFLD syndromes through quercetin, β-carotene and diosgenin.

Further, we identified 23 compounds of SSJZF which acted on 25 key therapeutic targets of NAFLD, such as Mapk14, MMP9, Myc, Bax. Xiao et al. (2017) reported that Mapk14 restricted the hepatic fat accumulation by antagonising JNK activation. Hwang et al. (2020) reported that hepatocyte-specific deletion of Mapk14 in HFD-induced fatty liver exacerbated steatosis and liver injury, indicating Mapk14 plays distinct roles in NAFLD. Zhang et al. (2019) indicated that macrophage Mapk14 promotes nutritional steatohepatitis through M1 polarisation. Recent studies have shown that MMP9 levels have been identified as a predictor of poor prognosis in patients with NAFLD (Coilly et al. 2019). Our data indicated MMP9 was a key target of SSJZF in the treatment of NAFLD. In addition, studies have shown that Myc activity was repressed by SIRT7 to suppress ER stress and prevent fatty liver disease (Shin et al. 2013), suggesting that Myc played an important role in NAFLD. A previous study showed that increasing the activation of BAX accelerates apoptotic death in severe hepatic steatosis (Liu et al. 2013). Blueberry combined with probiotics alleviates NAFLD via activation of JAK1/STAT3 signalling and inhibition of the apoptosis factor BAX (Zhu et al. 2018). Studies reported that transmembrane BAX inhibitor motif-containing 1 and Bax inhibitor-1 attenuated non-alcoholic steatohepatitis by a novel anti-inflammatory approach or limiting IREα signalling (Lebeaupin et al. 2018; Wree et al. 2018). Our results identified these hub genes as SSJZF potential targets on NAFLD were in accordance with previous studies to some extent. In-depth investigation of these targets may provide us novel therapies of NAFLD.

Numerous signalling pathways were screened based on the network pharmacology as well as RNA sequencing, including MAPK signalling pathway, TNF signalling pathway, and PI3K/Akt signalling pathway. Tumour necrosis factor (TNF) is a pivotal modulating component of the immune system, which regulates innate and adaptive immunity and implicates different inflammatory diseases such as tumour and inflammatory bowel disease (Lopetuso et al. 2018; Fischer et al. 2020). It has been reported that TNF played an important role in NAFLD progression, and NASH patients exhibited higher levels of TNF (Handa et al. 2017). Anti-TNF agents have been reported protective effects in NAFLD animal models including reduction of AST, ALT, hepatic inflammation and fibrosis (Koca et al. 2008). Another study has revealed that curcumin, an active ingredient of turmeric [*Curcuma longa* Linn, (Zingiberaceae)], could decrease TNF level in serum and improve NASH in patients (Saadati et al. 2019). These results suggested that TNF might be a potential target for NAFLD treatment. Moreover, the PI3K/Akt signalling pathway has been conserved to regulate and maintain appropriate cell growth, survival and metabolism (Barker et al. 2020), which is an important signalling pathway of insulin action and related to NAFLD progression (Zeng et al. 2014). We initially determined whether the PI3K/Akt signalling pathway was involved in the efficiency of SSJZF for NAFLD treatment, as previous studies have emphasised the significant role the PI3K/AKT pathway plays in process of NAFLD. The PI3K/Akt signalling pathway might modulate NAFLD progression (Liu et al. 2012; Matsuda et al. 2013). Akt can reduce the expression of fatty acid oxidation genes to regulate the process of hepatic glucose and lipid metabolism (Zeng et al. 2014). Previous studies also indicated the role the PI3K/Akt signalling pathway played in NAFLD treated with TCM. For example, Tangganjian decoction decreased triglyceride and total cholesterol, and increased the expression of PI3K and Akt in liver (Fan et al. 2018), as well as Shengmai-Yin and Ganmaidazao decoction, which could improve liver metabolism partially by activiting the PI3K/Akt pathway in type 2 diabetes mellitus with NAFLD (Sun et al. 2019). Our results were consistent with previous studies to some extent. It proved that SSJZF promoted activation of PI3K/Akt signalling, correlating with the evidence that the pathway alleviated hepatic lipogenesis and regulated lipid metabolism (Wang et al. 2014; Liao et al. 2018). Therefore, activation of the PI3K/Akt signalling pathway might be the mechanism of amelioration of NAFLD treated by SSJZF.

However, this study has several limitations. First, a lack of rescue experiment was performed to verify SSJZF improve hepatic lipid accumulation, hepatic fibrosis, and hepatic function injury in rats with NAFLD model by regulating PI3K/Akt pathway signalling. Second, the molecular mechanism of SSJZF regulation of PI3K/AKT pathway signalling has not been further clarified. Third, we screened out 23 chemical compounds as the potential effective ingredients of SSJZF by the comprehensive analysis of network pharmacology and mRNA-sequencing data mining, but high-performance liquid chromatography coupled with quadrupole time-of-flight mass spectrometry technique (HPLC-QTOF-MS) was not used to further identify these compounds in SSJZF and blood-absorbed compounds. In future study, these parts will be included in our experiments.

## Conclusions

In the present study, our data revealed that SSJZF ameliorated HLF-induced NAFLD via activating the PI3K/AKT pathway signalling. Importantly, we screened out 23 chemical compounds as the potential effective ingredients of SSJZF which acted on 25 targets of NAFLD by the comprehensive analysis of network pharmacology, mRNA-sequencing data mining and bioinformatics. This study not only provided theoretical basis for the application of SSJZF in clinical treatment of NAFLD, but also screened out some major compounds of SSJZF and their potential targets which will facilitate the development of new drugs for NAFLD.

## Author contributions

All authors made substantial contributions to the conception, study design, acquisition of data, analysis and interpretation of data; took part in drafting the article or revising it critically for important intellectual content; agreed to submit to the current journal; gave final approval of the version to be published; and agree to take responsibility and be accountable for the contents of the work.
